# Cu(C_3_H_3_N_3_S_3_)_3_ Adsorption onto ZnTiO_3_/TiO_2_ for Coordination-Complex Sensitized Photochemical Applications

**DOI:** 10.3390/ma15093252

**Published:** 2022-04-30

**Authors:** Ximena Jaramillo-Fierro, Karol Hernández, Silvia González

**Affiliations:** 1 Departamento de Química, Facultad de Ciencias Exactas y Naturales, Universidad Técnica Particular de Loja, San Cayetano Alto, Loja 1101608, Ecuador; sgonzalez@utpl.edu.ec; 2 Ingeniería Química, Facultad de Ciencias Exactas y Naturales, Universidad Técnica Particular de Loja, San Cayetano Alto, Loja 1101608, Ecuador; kyhernandez@utpl.edu.ec

**Keywords:** photosensitization, semiconductors, coordination-polymers, photocatalysis, adsorption, DFT

## Abstract

Currently, the design of highly efficient materials for photochemical applications remains a challenge. In this study, an efficient semiconductor was prepared, based on a coordination complex (Cu-TTC) of Cu(I) and trithiocyanuric acid on ZnTiO_3_/TiO_2_ (ZTO/TO). The Cu-TTC/ZTO/TO composite was prepared by the solvothermal method at room temperature. The structural, optical, and electrochemical characteristics, as well as the photocatalytic performance of the composite, were experimentally and computationally studied. The results show that the Cu-TTC/ZTO/TO composite efficiently extended its photoresponse in the visible region of the electromagnetic spectrum. The electrochemistry of the proposed tautomeric architecture (*s*-Cu-TTC) clearly reveals the presence of metal–ligand charge-transfer (MLCT) and π → π* excitations. The maximum methylene blue (MB) dye photodegradation efficiency of 95% in aqueous solutions was achieved under the illumination of simulated solar light. Finally, computational calculations based on the Density Functional Theory (DFT) method were performed to determine the electronic properties of the *s*-Cu-TTC tautomeric structure and clarify the adsorption mechanism of this complex on the surface (101) of both ZnTiO_3_ and TiO_2_ oxides. The results obtained allow us to suggest that the Cu-TTC complex is an effective charge carrier and that the Cu-TTC/ZTO/TO composite can be used efficiently for photochemical applications.

## 1. Introduction

In recent years, environmental pollution has become one of the most critical global concerns [1]. In fact, there is a wide variety of contaminants in water and wastewater today, which has attracted a lot of attention because of their potential environmental and health impacts. Among these contaminants are dyes produced mainly by the textile industry which are quite resistant to chemical oxidation treatments, and their toxicity makes biological degradation difficult, so discharges into the environment represent a very worrisome problem [2].

Methylene blue (MB) is one of the best well-known basic/cationic dyes and has been widely used in various industries. Due to the presence of multiple aromatic rings in its molecular structure, this dye is very stable and its biological degradation is very problematic, so the number of investigations on MB elimination has been continuously increasing [3]. Photocatalysis is one of the most used processes for the elimination of pollutants in the environment. Due to its high effectiveness and relatively low cost, this process has attracted extraordinary attention in the last two decades [4]. Photocatalysis occurs in the presence of natural or artificial light and requires the use of semiconductors, including metal oxides (TiO_2_, ZnO, WO_3_, Fe_2_O_3_) and chalcogenides (ZnS, CdS, CdSe) [5,6]. These compounds are characterized by different bandgap energies and oxidizing power and also by their ability to degrade organic pollutants [7].

Among semiconductors, zinc (Zn) and titanium (Ti) oxides have been widely exploited for various photodegradation applications because they are efficient, inexpensive, non-toxic, harmless, and exhibit chemical and thermal stability [8,9,10]. However, these oxides also have certain disadvantages, such as the high rate of carrier generation and recombination and the wide bandgap that only allows them to adsorb ultraviolet light [11,12]. Zn and Ti oxides can occur as binary (ZnO and TiO_2_), ternary (ZnTiO_3_, Zn_2_TiO_4_ and Zn_2_Ti_3_O_8_) or mixed compounds [13,14,15], all of which are attractive alternatives in various scientific and technological applications, for example, solar cells, gas sensors, pigments, photocatalysts, photoelectrochemical devices, UV protection materials, hydrogen generation, wastewater decontamination, etc. [16,17,18,19,20].

The great development of light-assisted technologies has motivated the investigation of electron transport materials (ITEM) [21,22,23,24], with a high quantum efficiency for practical photochemical applications under solar light (~300–800 nm) [25]. Therefore, several alternatives have been tested, the main ones being the coupling of photocatalysts and the use of dopants and sensitizers [26].

Semiconductor coupling has been demonstrated to be an interesting proposal to compensate for the disadvantages of the individual components. This proposal leads to more efficient charge separation, an extended lifetime of charge carriers, and enhanced interfacial charge transfer to adsorbed substrates [27,28]. Various semiconductors have been reported for the potential coupling of TiO_2_, including ReS_2_, SiO_2_, MoO_3_, CdS, MgO, WO_3_, SnO_2_, ZrO_2_, CuO, Fe_2_O_3_, and ZnO [29,30]. The features and compatibility of each coupling semiconductor are important for the physicochemical characteristics and stability of the integrated semiconductor. Each semiconductor significantly influences the surface charge of the material and, therefore, enhances or reduces its photocatalytic capacity [31]. In the doping procedure, fast charge recombination is delayed and visible light absorption is activated, leading to defective states in the bandgap [32]. In the former case, recombination is avoided and interfacial charge transfer is improved by trapping VB holes or CB electrons at defective sites. In the second case, electronic transitions from the defect states to the CB or from the VB to the defect state are permitted under forbidden sub-bandgap irradiation. Non-metal ions and metal ions are the two main groups of doping agents. In general, selective metals are desired as they have the ability to transfer electrons and reduce the energy level of the bandgap. Among the different metallic dopant elements, Cu, Au, and Ag have been shown to be effective dopants to improve the absorption of visible light [33].

On the other hand, several studies have shown that the photochemical efficiency of semiconductor materials can be improved by the use of sensitizing molecules. These sensitizers are adsorbed on semiconductors to improve their efficiency by inserting electrons between the conduction band of the semiconductor and the excited molecule. Sensitization performance generally depends on several aspects, including the energy level and molecular structure of the sensitizer [34,35].

Currently, the research on sensitizers is developing rapidly. This group of chemical structures includes various types, such as natural dye sensitizers, metal complex sensitizers, and metal-free organic sensitizers [36]. Among these, complexes based on transition metals are especially recognized as effective photosensitizers since they exhibit advantages including a long excited-state lifetime, as well as highly efficient light absorption and charge transfer from the metal to the ligand [37]. In addition, several studies have reported the high adhesion capacity of these complexes on semiconductor surfaces [38] through various adsorption modes, including the monodentate (M) mode, bidentate bridging (BB) mode, and bidentate chelating (BC) mode [39,40].

Likewise, other complexes based on transition metals, such as the so-called coordination polymers, have been the focus of numerous investigations due to their fascinating molecular architectures and potential technological applications [41,42]. A coordination polymer is a periodic array composed of metal ions that are bridged by organic ligands [28,43]. This type of molecular topology includes simple one-dimensional (1D) chains with small ligands, as well as large mesoporous frameworks [44,45]. In general, the formation process of coordination polymers proceeds spontaneously and is a self-assembly process [46].

Generally, the structural diversity of such polymers depends on several factors, such as the nature (hardness or softness, oxidation number) of the metal ion, the template agents, the metal–ligand ratio, the pH value, the counter-anion, the available number of coordination sites, and the various coordination modes generated by the organic ligands [47]. Regarding the strategies for synthesizing coordination polymers, the adequate selection of organic ligands or co-ligands according to their dimension, rigidity, and functional groups is essential for the assembly of manageable structures [48].

**Figure 1:**
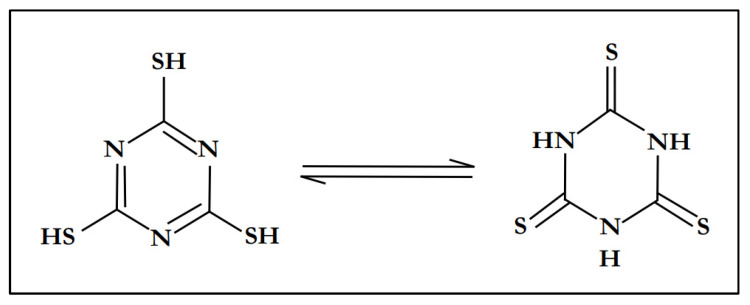
Tautomeric structures of trithiocyanuric acid.

Trithiocyanuric acid (C_3_H_3_N_3_S_3_), also referred to as trimercaptotriazine, is an interesting ligand with a symmetrical conformation characterized by the presence of sulfur and nitrogen atoms in a ring [49]. All of these are σ-donor atoms which can coordinate in a monodentate or bidentate fashion, and are capable of generating discrete complexes or polymeric/network structures [50]. In many complexes, trithiocyanuric acid (C_3_H_3_N_3_S_3_) usually exists as a tautomer, either as a thiol or as a thione [51]. Figure 1 shows the tautomeric structures of trithiocyanuric acid (C_3_H_3_N_3_S_3_).

Trithiocyanuric acid has three sets of N and S donor atoms, which can exhibit great coordination versatility. In fact, this molecule has the potential to coordinate with metallic elements through either the nitrogen or sulfur atoms, or even in a chelating *η*^2^-fashion via the SH-C=N group. Thus, the availability of multiple σ-donor atoms has allowed trithiocyanuric acid to behave as a bridging ligand in several reported polymer complexes, such as the Cu-TTC complex [Cu(C_3_H_3_N_3_S_3_)_3_] [52,53,54].

This paper reports the synthesis by the solvothermal method of the Cu-TTC/ZnTiO_3_/TiO_2_ composite for the efficient photodegradation of the methylene blue (MB) dye in aqueous solutions. In addition, the theoretical results of the interaction between the Cu-TTC complex and the surfaces (101) of the ZnTiO_3_ and TiO_2_ oxides are presented in order to clarify the experimental results obtained on the photocatalytic activity of the composite under solar light. Therefore, the feasibility of using Cu-TTC/ZnTiO_3_/TiO_2_ as an efficient alternative material for technological and environmental applications was experimentally and computationally demonstrated.

## 2. Materials and Methods

### 2.1. Materials

All reagents were purchased from commercial sources and used without further purification: Trithiocyanuric acid [C_3_H_3_N_3_S_3_] (Sigma-Aldrich, St. Louis, MO, USA, 95.0%), Copper(II) perchlorate hexahydrate [Cu(ClO_4_)_2_·6H_2_O] (Sigma-Aldrich, St. Louis, MO, USA, 98.0%), N,N-Dimethylformamide [(CH_3_)_2_-N-CHO] (Fisher Scientific, Waltham, MA, USA, 99.9%), Isopropyl alcohol [C_3_H_8_O] (Sigma Aldrich, St. Louis, MO, USA, ≥99.5%), Titanium(IV) isopropoxide [Ti(OC_3_H_7_)_4_] (Sigma Aldrich, St. Louis, MO, USA, 98%), Acetic acid [CH_3_COOH] (Fluka, 99,8%), Hydrogen chloride [HCl] (Fisher Scientific, Waltham, MA, USA, 37%), Cetyl-trimethyl ammonium chloride [C_19_H_42_NCl] (Sigma Aldrich, St. Louis, MO, USA, 25%), Hydrogen peroxide [H_2_O_2_] (Sigma Aldrich, St. Louis, MO, USA, 35%), Silver nitrate [AgNO_3_] (Sigma Aldrich, St. Louis, MO, USA, >99.8%), Nitric acid [HNO_3_] (Sigma Aldrich, St. Louis, MO, USA, 69%), Zinc acetate dihydrate [Zn(CH_3_COO)_2_·2H_2_O] (ACS, St. Louis, MO, USA, ≥98%), Methylene blue [C_16_H_18_ClN_3_S·xH_2_O] (Sigma Aldrich, St. Louis, MO, USA, ≥95%). Computational calculations were achieved using the Vienna Ab initio Simulation Package (VASP) version 6.0 (VASP Software GmbH, Vienna, Austria) and Gaussian version 09 (Gaussian, Inc., Wallingford, CT, USA) software packages.

### 2.2. Synthesis of ZnTiO_3_/TiO_2_ (ZTO/TO)

The ZnTiO_3_/TiO_2_ hybrid semiconductor was synthesized using a modified sol-gel method as we explained in previous studies [55]. A 70 v/v% solution of Ti(OC_3_H_7_)_4_ in C_3_H_8_O (Solution A) was prepared. Likewise, a solution formed by Zn(CH_3_COO)_2_·2H_2_O/H_2_O/C_3_H_8_O (Solution B) was prepared, using the stoichiometric for hydrolyzing Ti(OC_3_H_7_)_4_ and with a 50 v/v% C_3_H_8_O/H_2_O ratio. Then, solution B was slowly added to solution A to give a reaction system with a ZnO/TiO_2_ molar ratio of 1:3. This system was stirred until a white precipitate formed. The precipitate was dried at 60 °C for 24 h and calcined at 500 °C for 4 h. Finally, the products were cooled at room temperature.

### 2.3. Synthesis of Cu(C_3_H_3_N_3_S_3_)_3_ (Cu-TTC)

The complex of Cu(I) and trithiocyanuric acid was synthesized by a sedimentation method as we reported in previous studies [49]. In total, 1 mmol of Cu(ClO_4_)_2_·6H_2_O in DMF and 1 mmol of C_3_H_3_N_3_S_3_ in DMF were combined in a 100 mL beaker and mixed well by continuous agitation. A yellow-brown precipitate was formed immediately. The reaction was defined as finished when there was no further colour change; this usually occurred overnight, yet the solution was allowed to continue stirring for 2 to 3 additional days. After this time, the colour of the solid remained even after washing with cold methanol and drying in an oven at 60 °C.

### 2.4. Synthesis of Cu-TTC/ZTO/TO Composite

A [Cu(C_3_H_3_N_3_S_3_)_3_]/ZnTiO_3_/TiO_2_ (Cu-TTC/ZTO/TO) composite was synthesized by a routine solvothermal method. A proper amount of the Cu-TTC powder was dispersed in 50 mL of water and sonicated for 2 h to obtain a Cu-TTC suspension (25 g/L). Then, 20 mL of water, 10 mL of C_3_H_8_O and 10 mL of the Cu-TTC suspension were mixed and sonicated for 2 h. Later, the ZTO/TO powder (0.5 g) was added to the above suspension and stirred for 24 h at room temperature to obtain Cu-TTC/ZTO/TO particles suspended in the water/C_3_H_8_O solution. Next, this suspension in solution was placed into a 100 mL Teflon-lined autoclave and held at 100 °C for 12 h. The final Cu-TTC/ZTO/TO composite was obtained by precipitation, then washed and dried at 60 °C. A colour change of the samples from white to yellow-brown could be observed. 

### 2.5. Characterization of Cu-TTC/ZTO/TO Composite

The synthesized materials were characterized by powder X-ray diffraction and UV-Vis spectroscopy. X-ray diffraction (XRD) data were collected using a Bruker-AXS D8-Discover diffractometer (Bruker AXS, Karlsruhe, Germany) equipped with Cu Kα radiation (1.5406 Å). The patterns obtained from 5 to 70° in the 2θ range were compared with the ICDD database (International Centre for Diffraction Data, 2018) and the COD database (Crystallography Open Database, 2018) to identify the crystallographic phases. The UV-vis absorption spectra of the suspensions of the synthesized materials were recorded in the 200–1100 nm range using a fibre-coupled spectrometer (Thorlabs CCS200, Thorlabs Inc., Newton, NJ, USA).

### 2.6. Photocatalytic Activity

The photocatalytic performance of the catalytic materials was evaluated by the photodegradation of methylene blue dye under solar light [56]. A suspension was prepared by adding 250 mg of the composite material to an aqueous solution (100 mL) of methylene blue (MB) dye (25 mg/L).

The suspension was kept under stirring and in dark conditions for 30 min to allow adsorption–desorption equilibrium. Then, the suspension was irradiated with simulated solar light by a solar box (ATLAS, SUNTEST CPS+), equipped with an air-cooled 1500 W Xenon lamp (Atlas Material Testing Technology, Mount Prospect, IL, USA). Irradiance was set to 250 W/m^2^, and wavelengths of 300–800 nm were allowed to pass through.

Assays were run at pH = 7.0 with a controlled temperature of 25 °C. Residual MB concentrations were determined on a Jenway 7305 spectrophotometer (Bibby Scientific US Ltd, Manasquan, NJ, USA) at 663 nm. The photodegradation rate of the dye was estimated from the absorbance of the solution according to Beer Lambert’s law. Aliquots of the solution drawn at 30-min intervals were used for absorbance measurements. Before measurement, these aliquots were filtered through a 0.45 µm membrane to eliminate any particles that might disturb the measurement. Similarly, control experiments were developed to exclude any effect of photolysis due to light, using a methyl blue solution (without photocatalyst) that was also kept under stirring and in dark conditions for 30 min to allow for an adsorption–desorption equilibrium, and then was irradiated with solar light. All assays were performed in triplicate.

In addition, an experiment was designed to determine the regeneration property of the photocatalysts ZTO/TO and Cu-TTC/ZTO/TO. After completing one cycle of photodegradation of MB aqueous solution, the reaction system was allowed to stand for 1 h to precipitate the photocatalyst. Then, the supernatant liquid was removed from the system and 100 mL of fresh MB (25 mg/L) was added to the system to start the next photodegradation cycle. The regeneration experiment was developed for five consecutive cycles. Each cycle lasted 180 min under simulated solar light.

### 2.7. Computational Calculations

The calculations of geometrical parameters of the Cu-TTC molecule in the ground state were carried out with the Gaussian version 09 (Gaussian, Inc., Wallingford, CT, USA) software [57]. The structural properties were determined using a Density Functional Theory (DFT) formalism, with the RB3LYP functional (Becke’s three-parameter hybrid model using the Lee–Yang–Parr correlation) and Alhrich-TZV basis sets. The molecular electrostatic potential and HOMO-LUMO energies for Cu-TTC were evaluated at the same level of theory for the optimized structure [58].

On the other hand, the DFT calculations for Cu-TTC/ZTO and Cu-TTC/TO composites were performed using the VASP version 6.0 (VASP Software GmbH, Vienna, Austria) software [59]. The generalized gradient approximation (GGA) was used with the Perdew–Burke–Ernzerhof (PBE) exchange correlation function [60]. The electron–ion interactions were explained by the augmented plane wave (PAW) method [61]. The Kohn–Sham equations [62] were solved self-consistently until the energy variation between cycles was less than 10^−5^ eV. The cutoff energy to the plane waves was 500 eV. The adsorption of the Cu-TTC molecule on the surface of both ZnTiO_3_ and TiO_2_ was simulated using the following optimized parameters: hexagonal ZnTiO_3_ with a cell = 5.15 Å × 5.15 Å × 13.94 Å <90° × 90° × 120°> and tetragonal TiO_2_ with a cell = 3.82 Å × 3.82 Å × 9.70 Å <90° × 90° × 90°> [63]. The properties of the composites were calculated by sampling the first Brillouin zone using Monkhorst–Pack [64] *k*-point meshes of 3 × 2 × 1 and 1 × 3 × 1 for Cu-TTC/ZTO and Cu-TTC/TO, respectively. All atomic positions were fully relaxed until the respective forces were below 0.001 eV/Å. To improve the total energy convergence, the Gaussian smearing method with σ = 0.10 eV was utilized to band occupations. All calculations were non-spin polarized. For the design and visualization of the respective molecular models, the BioVia Material Studio, version 5.5 (BioVia, San Diego, CA, USA) software, was used.

The bulk of both ZTO and TO crystals was cleaved at the stable surface (101) [65,66,67] in order to study Cu-TTC adsorption. The slab model of ZTO (101) consisted of a supercell p(2 × 3), with 36 Zn atoms, 36 Ti atoms, and 108 O atoms, while the slab model of TO (101) consisted of a supercell p(3 × 3) with 168 Ti atoms and 336 O atoms. In addition, a vacuum of 20 Å was applied for both ZTO and TO surface models. The values for the surface energies (γ_s_) of the ZTO and TO structures with a vacuum distance of 20 Å were calculated in 0.076 eV/Å^2^ (7.30 kJ/Å^2^) and 0.062 eV/Å^2^ (5.98 kJ/Å^2^), respectively, using the following expression [68]:

(1) $$\gamma_{s}=\frac{\left(E_{\mathrm{slab}}-n\times {\;E}_{\mathrm{bulk}}\right)}{2A}$$

where E_slab_ is the total energy of the slab material (eV), E_bulk_ is the total energy of the bulk material (eV), *n* is the number of atoms included in the slab, and A is the surface area (Å^2^). On the other hand, the adsorption energy (ΔE_ads_) of the Cu-TTC molecule on the surface of both ZTO and TO oxides was calculated by the following expression [69]:

(2) $$\Delta E_{\mathrm{ads}}=E_{\mathrm{Cu}-\mathrm{TTC}/\mathrm{oxide}}-E_{\mathrm{oxide}}-E_{\mathrm{Cu}-\mathrm{TTC}}$$

where E_Cu-TTC/oxide_ is the energy of the supersystem formed by the adsorbed molecule on the surface (eV), E_oxide_ is the energy of the clean oxide (eV), and E_Cu-TTC_ is the energy of the isolated molecule in vacuum (eV).

## 3. Results

### 3.1. Characterization of the Samples

#### 3.1.1. XRD Analysis

**Figure 2:**
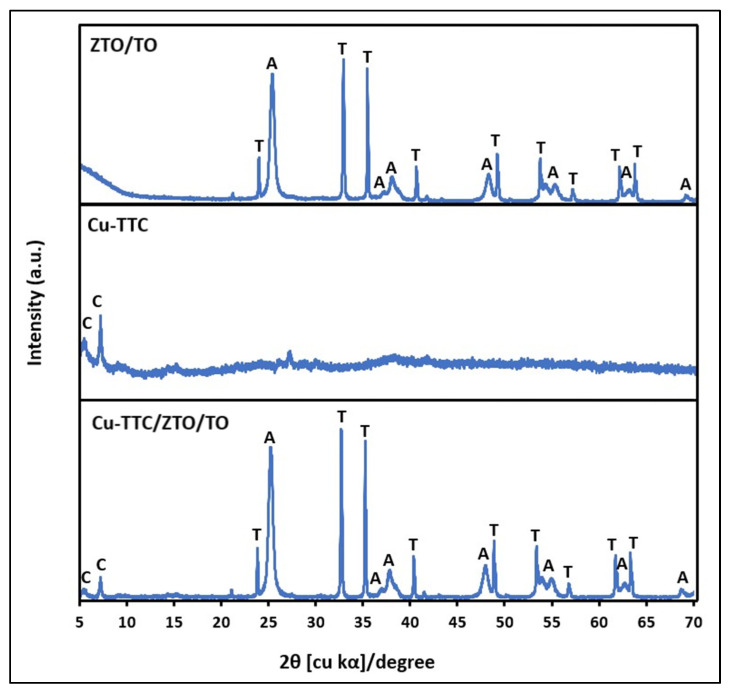
XRD of ZTO/TO, Cu-TTC and Cu-TTC/ZTO/TO. C: Cu-TTC; T: ZnTiO_3_; A: TiO_2-a_.

Figure 2 shows the XRD patterns of the ZTO/TO, Cu-TTC and Cu-TTC/ZTO/TO powder materials. The characteristic peaks of ZnTiO_3_ (ZTO) were shown at 2θ∼32.70° and 35.24° according to the standard COD card No. 00-026-1500. Likewise, the characteristic peak of the anatase (TO) phase was presented at 25.32° according to the standard COD card No. 01-071-1168. Furthermore, there are two peaks at 2θ∼5.54° and 7.21° corresponding to the presence of Cu-TTC complex according to the standard COD card No. 00-402-1455 and COD card No. 00-720-8980, respectively. The relative amount of the crystallographic phases ZTO and TO in ZTO/TO was estimated to be 47% and 53%, respectively, while the relative amount of the crystallographic phases ZTO, TO and Cu-TTC in the composite was estimated to be 37%, 41% and 22%, respectively.

The crystallite dimensions of the synthesized materials were calculated based on the main peak using the Scherrer equation [70]:

(3) $$A=\frac{K\lambda }{\beta \;\cos\theta }$$

where K is the shape factor (here, K = 0.89), λ is the wavelength of the X-ray beam used (λ = 0.15405 nm), θ is the Bragg angle, and β is the full width at half maximum (FWHM) of the X-ray diffraction peak. The mean size of the crystallites of the Cu-TTC, ZTO and TO phases in the Cu-TTC/ZTO/TO composite was 22.5 (±3.4), 119.7 (±3.6) and 16.5 (±3.7) nm, respectively. The crystallite dimension and relative amount of the crystallographic phases in the composite were estimated by adopting the analysis function DIFFRAC.EVA V.4.0 (Bruker AXS, Karlsruhe, Germany) software.

#### 3.1.2. UV-Vis Spectroscopy

**Figure 3:**
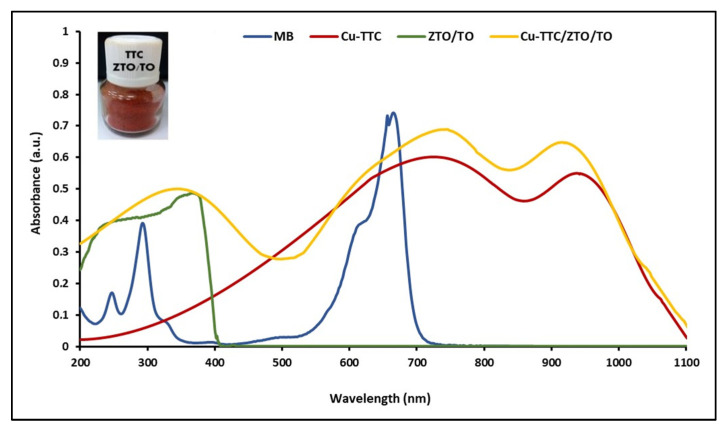
UV-Vis absorption spectra of MB, ZTO/TO, Cu-TTC and Cu-TTC/ZTO/TO.

UV-Vis electronic absorption spectra of the MB, ZTO/TO, Cu-TTC and Cu-TTC/ZTO/TO were recorded in the 200–1100 nm range at room temperature in solutions using 1 cm path-length quartz cells. Figure 3 shows in order the maximum absorption peaks for ZTO/TO, MB, Cu-TTC and Cu-TTC/ZTO/TO at 390, 663, 725 and 745 nm, respectively.

**Figure 4:**
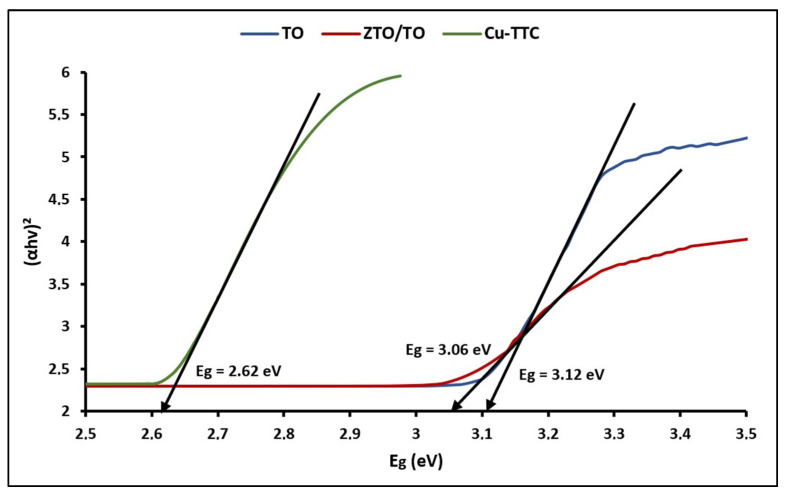
Plots of (αhv)^2^ vs. E_g_ of TO, ZTO/TO and Cu-TTC.

Figure 4 shows the plots of (αhv)^2^ versus hv for calculating bandgap energy (E_g_) using the expression [71]:

(4) $$E_{g}=\frac{1240}{\lambda }$$

where E_g_ is the bandgap energy in the electron volts (eV) and λ is the represents the lower cutoff wavelength in nanometer (nm).

The bandgap (E_g_) energy values were 3.12, 3.07 and 2.62 eV for TO, ZTO/TO and Cu-TTC, respectively. According to the literature, the E_g_ value estimated for ZTO/TO can be related to the direct bandgap of ZTO [72].

In addition, the Mulliken electronegativity theory was used to estimate the potentials of the VB and CB of Cu-TTC, TO and ZTO [73]:

(5) $$E_{\mathrm{CB}}=\chi -E_{c}-0.5E_{g}$$

(6) $$E_{\mathrm{VB}}=E_{\mathrm{CB}}+E_{g}$$

where E_CB_ and E_VB_ are the CB edge potential and VB edge potential, respectively, E_g_ is the bandgap energy of the compounds, E_c_ is the energy of free e- on the hydrogen scale (~4.5 eV), χ is the electronegativity of the compounds TO (χ = 5.8) [74], ZTO (χ = 4.0) [12], and Cu-TTC (χ = 6.3). The electronegativity of Cu-TTC was estimated by Gaussian calculations using a DFT formalism/RB3LYP method with the Alhrich/TZV basis sets. According to the above equations, the E_CB_ and E_VB_ for Cu-TTC, TO and ZTO were (+0.44, +3.06), (−0.26, +2.86) and (−2.03, +1.03) eV, respectively.

### 3.2. Photocatalytic Activity

**Figure 5:**
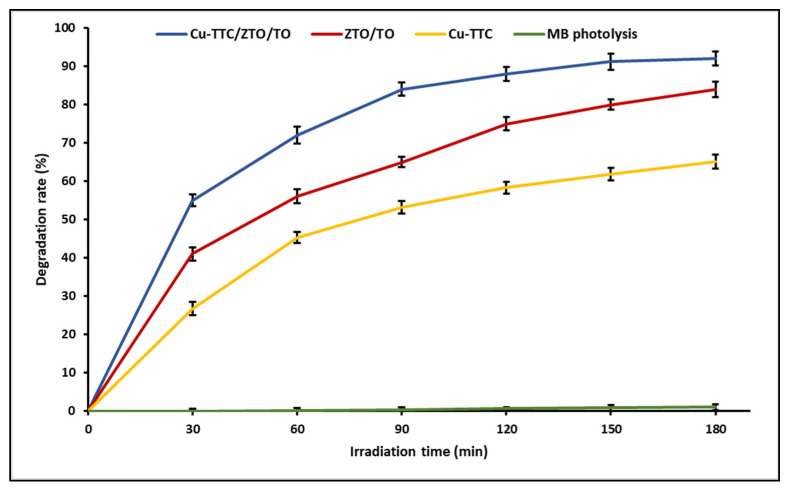
Photocatalytic degradation activity of Methylene Blue (MB) for photocatalyists Cu-TTC/ZTO/TO, ZTO/TO and Cu-TTC.

Photocatalysis allows efficient degradation of organic substances due to the great oxidizing capacity that photocatalysts acquire when they are irradiated by light. In a previous study, we determined the photocatalytic activity of ZTO/TO by the decomposition of MB dye in an aqueous solution. In this study, the photocatalytic activity of the Cu-TTC complex and the photocatalysts ZTO/TO and Cu-TTC/ZTO/TO was tested using solar light. Figure 5 shows that the Cu-TTC/ZTO/TO composite had higher photocatalytic activity than Cu-TTC and ZTO/TO. On the other hand, the effect of photolysis on dye degradation was negligible in control experiments (without photocatalyst), obtaining only around 1% degradation during the evaluation time.

In general, the photocatalytic reactions kinetics of MB dye, as well as other organic compounds, can be described by rationalized pseudo-first-order decomposition kinetics in terms of the Langmuir–Hinshelwood model, using the following expression [75,76]:

(7) $$\frac{C}{C_{0}}={\;e}^{-k_{\mathrm{app}}t}\;$$

where C is the reagent concentration at time t (mg/mL), C_0_ is the initial reagent concentration (mg/mL), k_app_ is the apparent rate constant (min^−1^), and t is the irradiation time (min).

**Figure 6:**
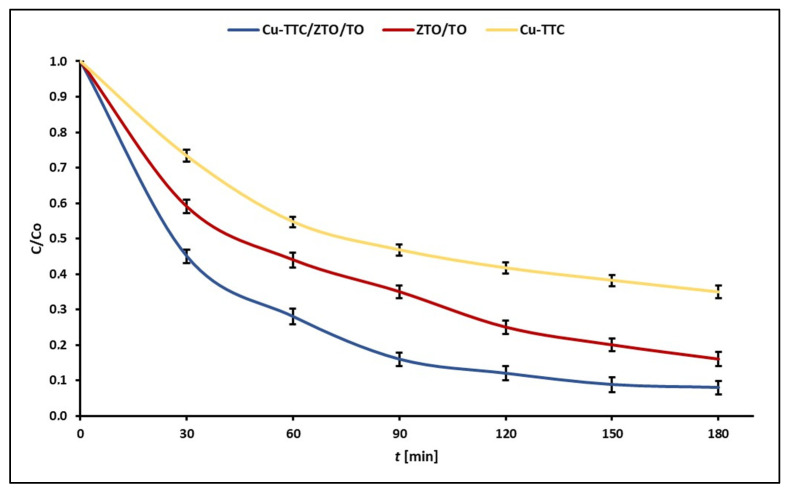
Evolution of experimental points of C/C_0_ during the photocatalytic degradation of MB dye for the photocatalysts Cu-TTC/ZTO/TO, ZTO/TO and Cu-TTC.

Figure 6 shows the experimental points of C/C_0_ at each reaction time for the photodegradation of MB dye for the photocatalysts Cu-TTC/ZTO/TO, ZTO/TO and Cu-TTC.

The values of apparent rate constants k_app_ for the photocatalysts Cu-TTC/ZTO/TO, ZTO/TO and Cu-TTC are shown in Table 1. These data show that the highest pseudo-first-order rate constant was obtained for Cu-TTC/ZTO/TO composite.

**Figure 7:**
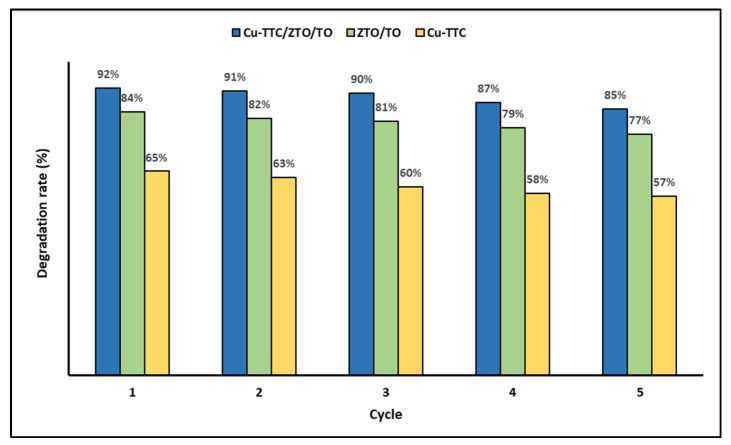
Degradation percentage of MB dye for five photocatalytic cycles.

In addition, the recyclability of the photocatalysts was evaluated as an important issue for their application on a large scale. Figure 7 shows the photodegradation efficiency of the photocatalysts Cu-TTC, ZTO/TO and Cu-TTC/ZTO/TO for five successive cycles.

In Figure 7, it is evident that the percentage of MB photodegradation decreases slightly as the number of cycles increases. However, in the fifth cycle, the synthesized materials can still efficiently photodegrade the dye in an aqueous solution.

### 3.3. Computational Calculations

#### 3.3.1. Optimization of the Cu-TTC Structure

**Figure 8:**
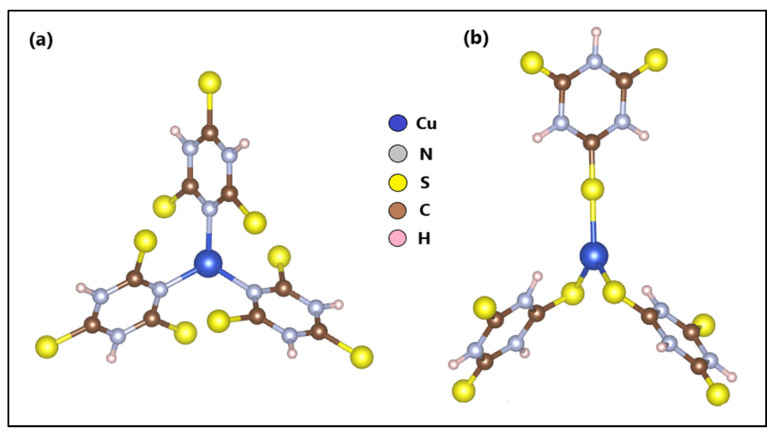
Optimized structures of (**a**) *n*-Cu-TTC and (**b**) *s*-Cu-TTC.

The optimization and calculation of the electronic structure of ZTO and TO were reported in a previous study. On the other hand, based on another previous study, where we reported the *n*-Cu-TTC tautomeric complex [49] (Figure 8a), the *s*-Cu-TTC tautomeric complex was constructed with the BioVia Material Studio, version 5.5 (Figure 8b) and optimized using a DFT formalism/RB3LYP method with the Alhrich/TZV basis sets.

The Gaussian calculations summaries for both tautomeric structures are compared in Table 2.

#### 3.3.2. Frontier Molecular Orbitals (HOMO-LUMO) of the Cu-TTC Structure

The HOMO (highest occupied molecular orbitals) and LUMO (lowest unoccupied molecular orbitals) energies and the electronegativity were estimated with the same level of theory and the results obtained are listed in Table 3.

**Figure 9:**
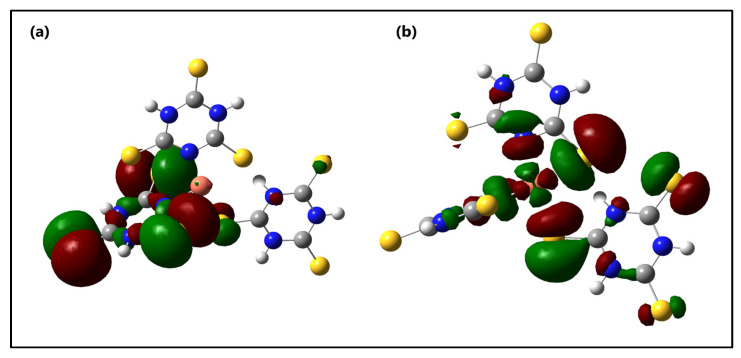
Molecular orbital surfaces for the (**a**) HOMO and (**b**) LUMO of the *s*-Cu-TTC.

The distribution and energy levels of the frontier molecular orbitals of the *s*-Cu-TTC tautomer are shown in Figure 9.

#### 3.3.3. Molecular Electrostatic Potential of the Cu-TTC Structure

**Figure 10:**
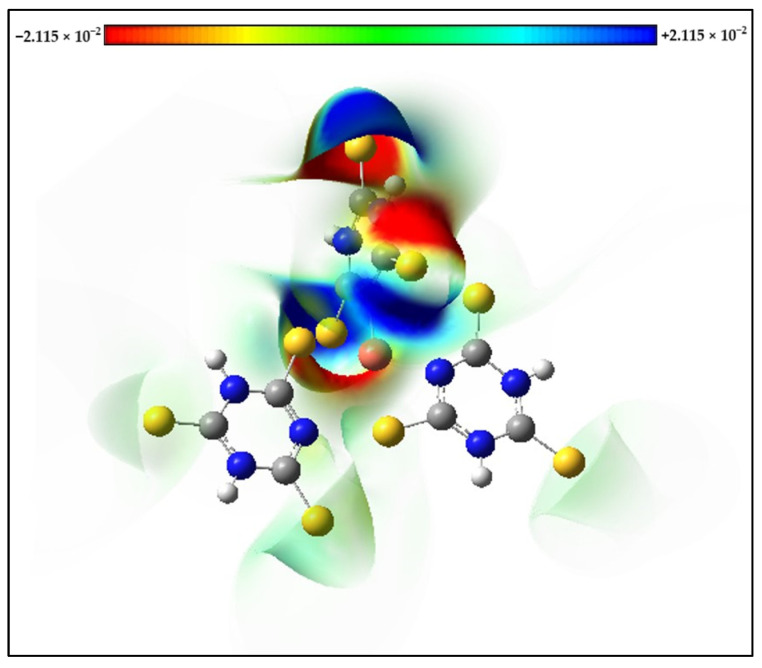
Molecular Electrostatic Potential (MEP) maps of the *s*-Cu-TTC.

Molecular Electrostatic Potential (MEP) maps of the *s*-Cu-TTC complex were calculated with the optimized geometry. The positive (blue) regions in the MEP are associated with nucleophilic reactivity and were located mainly over the N atoms from one of the ligands, while the negative (red) regions in the MEP are associated with electrophilic reactivity and were located mainly over the S atoms from the same ligand (Figure 10).

#### 3.3.4. Adsorption of the Cu-TTC on the ZnTiO_3_ (ZTO) and TiO_2_ (TO) Surfaces

**Figure 11:**
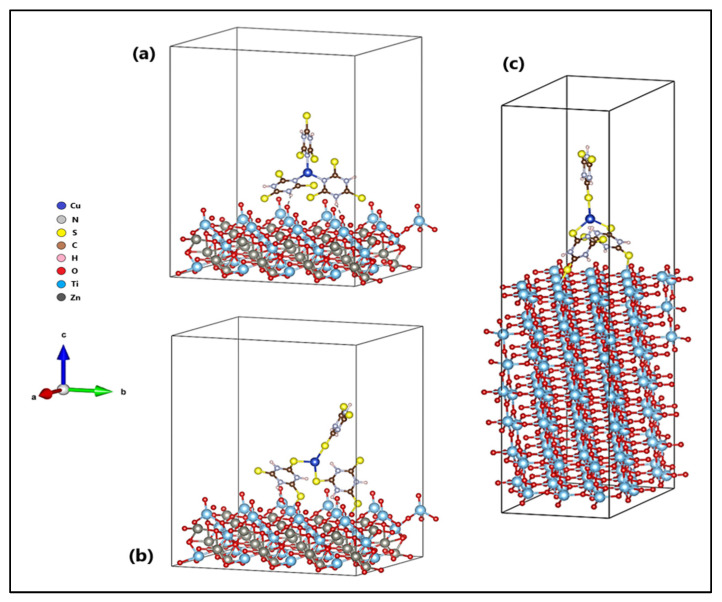
Cu-TTC molecule in (**a**) *n*-Cu-TTC and (**b**) *s*-Cu-TTC tautomeric form on the ZnTiO_3_ surface, and (**c**) *s*-Cu-TTC tautomeric form on the TiO_2_ surface.

The orientation of the Cu-TTC molecule on the surfaces (101) of ZTO and TO are shown in Figure 11. Figure 11b shows the *n*-Cu-TTC tautomeric form on the ZTO surface, while Figure 11a,c shows the *s*-Cu-TTC tautomeric form on the ZTO and TO surfaces, respectively. The adsorption of the *s*-Cu-TTC molecule on the ZTO surface (E_ads_ = −3.07 eV) was more energetically favoured than in *n*-Cu-TTC (E_ads_ = −1.69 eV). From these results, we studied the adsorption of *s*-Cu-TTC only on the surface (101) of TO, to establish a comparative study between both semiconductors. The results showed that the adsorption of the *s*-Cu-TTC molecule on the surface (101) of TO (E_ads_ = −1.21 eV) was less favourable than on the surface (101) of ZTO.

**Figure 12:**
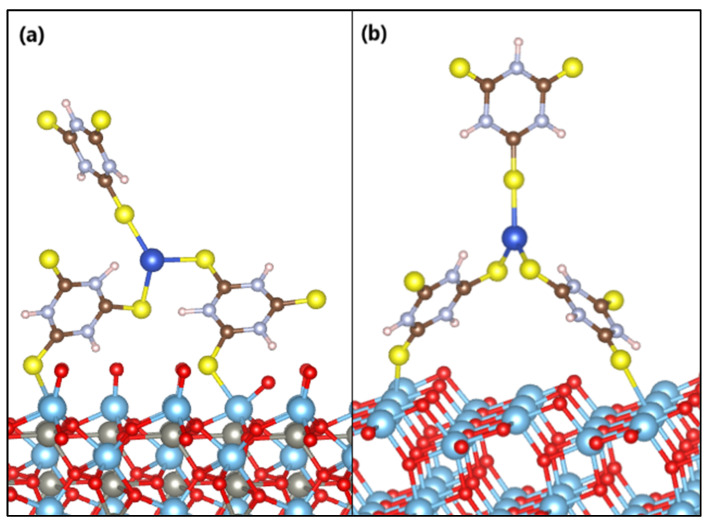
Anchoring modes of the *s*-Cu-TTC molecule on the surface of (**a**) ZnTiO_3_ and (**b**) TiO_2_.

The anchoring models of the *s*-Cu-TTC molecule on the ZTO and TO surfaces are displayed in Figure 12. Adsorption of the Cu-TTC complex on the ZTO and TO surfaces arises in a bidentate adsorption model with one sulfur atom from each surface-facing triazine ring bound to the nearest titanium atom.

The estimated adsorption energy value suggests that the Cu-TTC molecule is strongly adsorbed on the ZTO surface. The average distances from the sulfur atoms of the Cu-TTC molecule (S_Cu-TTC_) to the titanium atoms of the surface plane of ZTO (Ti_ZTO_) are S_Cu-TTC_-Ti_ZTO_ = 2.58 Å and S_Cu-TTC_-Ti_ZTO_ = 2.55 Å. Furthermore, the adsorption energy value of the Cu-TTC molecule on the TO surface suggests a weaker interaction than on the ZTO. The average distances from the sulfur atoms of the Cu-TTC molecule (S_Cu-TTC_) to the titanium atoms of the surface plane of TO (Ti_TO_) are S_Cu-TTC_-Ti_TO_ = 2.72 Å and S_Cu-TTC_-Ti_TO_ = 2.69 Å. The Ti–S bond distances calculated are very similar to those reported in the literature [77].

In order to further understand the chemical nature of the S-Ti bonds formed in the Cu-TTC/ZTO and Cu-TTC/TO composites, the population analyses were estimated by the AIM method proposed by Bader and the results are listed in Tables S1 and S2. For Cu-TTC/ZTO, the net charge of Ti (+2.5*e*) is +1.5*e* smaller than its +4*e* formal charge, whereas the net charge of S (+1.0*e*) is +5.0*e* smaller than its +6*e* formal charge. For Cu-TTC/TO, the net charge of Ti (+2.6*e*) is +1.4*e* smaller than its +4*e* formal charge, whereas the net charge of S (+1.2*e*) is +4.8*e* smaller than its +6*e* formal charge. In both cases, the donation of electronic charge from the molecule to the surface is evident.

## 4. Discussion

### 4.1. Characterization of the Samples

#### 4.1.1. XRD Analysis

Firstly, the structural identification of the Cu-TTC/ZTO/TO composite was performed using XRD. Figure 2 shows the diffraction pattern of the composite, in which the appearance of two peaks at low angles 2θ corresponding to the Cu-TTC phase was evident. The presence of these peaks revealed the incorporation of the Cu-TTC coordination complex into the ZnTiO_3_/TiO_2_ heterostructure, which we reported in previous studies. The average size of the ZTO and TO crystallites in the ZTO/TO hybrid semiconductor was similar to that calculated for the ZTO and TO phases of the composite. Therefore, the addition of Cu-TTC did not affect the crystal structure of ZTO/TO. In addition, no preferential orientations were observed.

#### 4.1.2. UV-Vis Spectroscopy

An efficient photochemical sensitizer should be strongly anchored to the semiconductor surface, and display intense absorption in the visible and the infrared region of the spectrum [65]. In this study, electronic absorption spectra of the heterogenous photocatalyst ZTO/TO in its free form and after adsorbing the Cu-TTC complex were investigated. The λ_max_ is an essential criterion for the suitability of this metal–organic system to be considered as a photochemical sensitizer. The wavelength of maximum absorption of the Cu-TTC/ZTO/TO composite was 745 nm, showing a bathochromic shift with respect to the maximum absorption at 390 nm of the free ZTO/TO photocatalyst. The molar extinction coefficient (ε) to the Cu-TTC/ZTO/TO composite at a concentration of 2.50 × 10^−9^ mM was calculated to be around 23,200 M^−1^ cm^−1^. Given the strong absorbance observed in the spectrum, it was not possible to identify any Laporte forbidden d-d transitions, which were probably buried under other adsorptions because of their low extinction coefficient. As with the Laporte forbidden transitions, the observation of any n → π* transition was not obvious because of their very small extinction coefficient [49].

Based on the proposed geometry of the Cu-TTC complex, the strong absorption band of the Cu-TTC/ZTO/TO composite in the visible region is believed to be due to the metal–ligand charge transfer (MLCT) state as well as π → π* excitations of the Cu-TTC complex [78,79].

### 4.2. Photocatalytic Activity

The photocatalytic activity of the Cu-TTC/ZTO/TO composite was determined by degradation under solar light irradiation of Methylene Blue dye in aqueous solutions. According to the literature, photoexcited catalysts can degrade organic molecules, such as MB, due to the movement of photogenerated electron–hole pairs (e^−^/h^+^) on the catalyst surface. The e^−^ moving toward the catalyst surface reacts with O_2_ in the solution to produce ˙OH and ˙O_2_ radicals. These radicals oxidize the MB molecule achieving the loss of colour and photodegradation of this dye [80].

The results obtained in the present investigation suggest that the coupled semiconductor ZTO/TO has a great photocatalytic performance. These results are in agreement with those reported in the literature [81]. Furthermore, the Cu-TTC/ZTO/TO composite displayed a significant improvement in photocatalytic activity compared to ZTO/TO and Cu-TTC. The composite possibly displayed a high photocatalytic activity since the incorporation of Cu-TTC contributes to an efficient separation of photo-generated e^−^/h^+^ pairs between ZnTiO_3_ and TiO_2_ [82]. The data in Table 1 show that the kinetic constant calculated according to the Langmuir–Hinshelwood model for the MB dye photodegradation reaction is higher when using Cu-TTC/ZTO/TO compared to the ZTO/TO photocatalyst and Cu-TTC complex. This supports the high photocatalytic activity shown by the synthesized composite.

**Figure 13:**
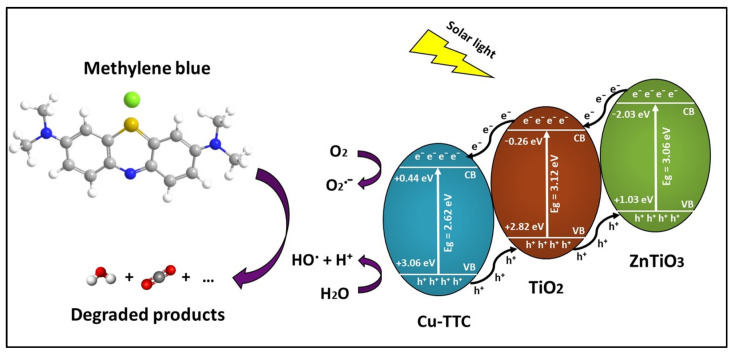
Schematic of electron–hole separation and transportation on the interface Cu-TTC/ZnTiO_3_/TiO_2_. heterojunction.

When the Cu-TTC/ZTO/TO is irradiated by solar light, the e^−^ of Cu-TTC, which is anchored on the surface of ZTO/TO, may be excited from the ground state. The photoexcited state of Cu-TTC which is generated by the light absorption probably corresponds to the MLCT excitations, as well as π → π* transitions from the triazine rings of the Cu-TTC. As a result, e^−^ can move freely on the surface, promoting better photoelectrochemical and photocatalytic performance of the ZTO/TO photocatalyst under solar light. Figure 13 describes the proposed photocatalytic mechanism of the Cu-TTC/ZTO/TO heterojunction. From this figure, it is evident that the energy values of the conduction band (CB) and valence band (VB) decreased in the following order Cu-TTC > TO > ZTO. As a result, the excited electrons from the CB of ZTO jumped to the CB of TO and then to the CB of Cu-TTC, while the holes generated by the VB of Cu-TTC were transferred to the VB of TO and then to the VB of ZTO, resulting in a suitable energy cascade to improve the mobility of electrons in the composite and reduce the interfacial recombination of electron–hole pairs, promoting an enhanced photocatalytic effect [83,84].

From Figure 13, it is clear that Cu-TTC could also offer an alternative pathway to generate reactive oxygen species (ROS), which are fundamental for photocatalytic processes. In fact, the Cu(I) form Cu-TTC could interact with molecular oxygen O_2_ under solar light irradiation and motivate its activation. The molecular oxygen activation would allow the photoproduction of free or coordinated superoxo species, Cu(II)-O_2_˙^−^ (reaction R1), and then H_2_O_2_ (reaction R2) and ·OH (reactions R3 and R4) [85]. These reactive oxygen species (ROS), in particular ·OH, are necessary for achieving the effective photodegradation of the MB dye (reaction R5). The following reactions represent the possible contribution mechanism of Cu-TTC for the generation of ROS.

O_2_˙^−^ + H^+^ → HO_2_ (R1)

2 HO_2_˙ → O_2_ + H_2_O_2_ (R2)

Cu(I) + H_2_O_2_ → Cu(II) + ·OH + OH^−^ (R3)

H_2_O_2_ + O_2_˙^−^ → ·OH + OH^−^ + O_2_ (R4)

OH or h^+^ + MB → degradation (R5)

On the other hand, in the regeneration tests, it was shown that on average the deactivation of the Cu-TTC/ZTO/TO, ZTO/TO and Cu-TTC photocatalysts did not exceed 10% after five consecutive cycles, suggesting that these photocatalysts could have important environmental applications for the photodegradation of methylene blue dye in aqueous systems.

Although the experiments conducted in the present study demonstrated the photodegradation of MB in an aqueous solution, this dye could also act in the first instance as an antenna and contribute to the light absorption efficiency of the sensitized semiconductor Cu-TTC/ZTO/TO. In this way, the photocatalytic system would be constituted by the sensitizer (P) and antenna (A) components covalently bonded to each other and adsorbed through the sensitizer on the surface of the third component, the semiconductor (S). In this proposed photocatalytic system, the antenna (A) has the function of strongly absorbing the incident light and efficiently transferring the electronic energy to the sensitizer, which then injects a charge into the semiconductor [86]. In this way, both the light directly absorbed by the sensitizer Cu-TTC and that absorbed by the MB antenna can be used for the effective injection of charge in the hybrid semiconductor ZTO/TO.

### 4.3. Computational Calculations

Frontier molecular orbitals, HOMO and LUMO, are the principal orbital involved in chemical reactions and are also employed for estimating the mainly reactive position in π-electron systems. The HOMO energy reveals the ability of electron-donating orbitals, while the LUMO reveals electron-accepting ability. Likewise, the energy gap between HOMO and LUMO reveals molecular chemical stability [87,88]. The low bandgap values listed in Table 3 for both tautomeric structures help to support the eventual charge transfer interactions that occur within the molecule.

From Figure 9, it is suggested that there exists an efficient electron transfer between ligands, from an S atom of the HOMO to the other S atom of the LUMO when an electronic transition occurs. The HOMO for the computed system exhibits very little metal character and it is localized mainly at the S atoms and the triazine ring from one of the ligands, whereas the LUMO is found to possess a strong metal character, and is located mainly at the S atoms from other, different ligand rings. This result suggests that there is an evident intramolecular charge transfer (ICT) from HOMO^0^ to LUMO^0^, which occurs through a π-conjugated path. This intramolecular charge transfer is common in low valence metal complexes with accessible orbitals from the ligands [89].

Our theoretical results indicated that the adsorption of the *s*-Cu-TTC tautomer was more favoured than that of the *n*-Cu-TTC tautomer on the ZnTiO_3_ surface. In addition, the *s*-Cu-TTC molecule was adsorbed on the ZnTiO_3_ surface (101) with higher negative energy (E_ads_ = −3.07 eV) than on the TiO_2_ surface (101) (E_ads_ = −1.21 eV). According to the optimized configurations, *s*-Cu-TTC adsorption occurs on the ZnTiO_3_ and TiO_2_ surfaces (101) in a bidentate coordination mode through S-Ti bonds, which produces more stable adsorption with more exotherm adsorption energy. Therefore, in this study, it was demonstrated that both the adherence strength and the mode of anchoring of the molecule on the oxide surface are strongly influenced by the type of tautomeric structure (*n*-Cu-TTC or *s*-Cu-TTC).

In the literature, no studies of Cu-TTC adsorption on semiconductors were found; consequently, in Table 4, the results obtained in the present investigation are compared with those results reported in the literature for the adsorption of other molecules of ZnTiO_3_ (101) and TiO_2_ (101). All the results shown in this table were obtained by computational calculations using the general gradient approximation (GGA) applying Perdew–Burke–Ernzerhof (PBE) as the correlation functional.

Regarding the population analyses estimated by the AIM method, the results evidenced the donation of electronic charge of the Cu-TTC complex to the surface of both ZTO and TO. Since the value of charges on the different bonds can suggest the covalent and ionic characteristics of these bonds, we conclude that the S–Ti bonds are certainly covalent for both Cu-TTC/ZTO and Cu-TTC/TO. These results agree with those reported in the literature for similar architectures [90].

## 5. Conclusions

The analytical and physicochemical analyses provided important evidence that supports the proposed structure of the Cu-TTC/ZTO/TO composite. The coordination sphere of the *s*-Cu-TTC tautomer can be best described as trigonal bipyramidal. In the Cu-TTC/ZTO/TO composite, two triazine rings act as bidentate ligands which coordinate to a Ti atom through their S atoms. The optimized geometry was calculated employing a hybrid DFT/RB3LYP method with Alhrich-TZV levels of theory for the first time. Furthermore, the calculated HOMO and LUMO energies and energy gap analysis suggest that there is an intramolecular donor–acceptor charge transfer that substituted triazine ligands through a π-conjugated path. This transition (HOMO^0^→LUMO^0^) is predicted as a π-π* transition. Due to these transitions, the Cu-TTC/ZTO/TO composite was found to be highly efficient (92%) when compared to ZTO/TO (84%) in photodegrading MB dye from aqueous solutions.

In addition, the purpose of this comparative study was to apply molecular simulation to address unresolved issues associated with the adsorption mechanism of Cu-TTC on both ZnTiO_3_ and TiO_2_. DFT calculations of *s*-Cu-TTC adsorption on the surface (101) of both ZnTiO_3_ and TiO_2_ denoted that adsorption on ZnTiO_3_ was stronger than on TiO_2_.

Finally, we supported the viability of using Cu-TTC as a possible ROS-generating photosystem, as well as a photosensitizer of the heterogenous photocatalyst ZnTiO_3_/TiO_2_. In this way, the Cu-TTC/ZTO/TO composite represents an efficient alternative material for various technological and environmental applications.

## Figures and Tables

**Table 1:** Calculated k_app_ values for the photodegradation of MB.

	ZTO/TO	Cu-TTC/ZTO/TO	Cu-TTC
k_app_ (min^−1^)	9.8 × 10^−3^ (±5.1 × 10^−5^)	1.4 × 10^−2^ (±4.1 × 10^−4^)	5.6 × 10^−3^ (±3.9 × 10^−4^)
R^2^	0.96	0.95	0.93
χ^2^	1.5 × 10^−3^	2.3 × 10^−2^	1.8 × 10^−3^

**Table 2:** Gaussian calculations summary.

Specifications	Results	Results
File Name	*n*-Cu-TTC	*s*-Cu-TTC
Calculation Method	RB3LYP	RB3LYP
Basis Set	TZV	TZV
Charge	0	0
Spin	Singlet	Singlet
E_(UB3LYP)_ (a.u.)	−6063.7277	−6063.7287
RMS Gradient Norm (a.u.)	0.00000911	0.00000782
Imaginary Freq	0	0
Dipole Moment (Debye)	2.4997	3.0174
Point Group	C1	C1

**Table 3:** Frontier orbital energies and electronegativity.

	*n*-Cu-TTC	*s*-Cu-TTC
E_HOMO_ (eV)	−6.67	−6.71
E_LUMO_ (eV)	−5.83	−5.89
Electronegativity	6.25	6.30

**Table 4:** Calculated adsorption energy values of s-Cu-TTC on ZnTiO_3_ and TiO_2_ and other values of several dyes reported in the literature.

Adsorbent (Surface)	Dye	Software Used	Adsorption		References
			eV	kJ/mol	
TiO_2_ (101)	R4-BT	VASP	−1.40	−135.46	[58]
TiO_2_ (101)	R4-F2BT	VASP	−1.39	−134.50	[58]
TiO_2_ (101)	R4-BO	VASP	−1.39	−134.50	[58]
TiO_2_ (101)	R6-Bz	VASP	−1.40	−135.46	[58]
TiO_2_ (101)	R6-BT	VASP	−1.38	−133.53	[58]
TiO_2_ (101)	R6-F2BT	VASP	−1.37	−132.56	[58]
TiO_2_ (101)	R6-B0	VASP	−1.37	−132.56	[58]
TiO_2_ (101)	R6-Bz	VASP	−1.38	−133.53	[58]
TiO_2_ (101)	MB	VASP	−0.12	−11.61	[63]
TiO_2_ (101)	*s*-Cu-TTC	VASP	−1.21	−116.95	This study
ZnTiO_3_ (101)	TPA-1	CASTEP	−1.41	−136.39	[66]
ZnTiO_3_ (101)	TPA-2	CASTEP	−1.63	−157.47	[66]
ZnTiO_3_ (101)	TPA-3	CASTEP	−5.82	−561.33	[66]
ZnTiO_3_ (101)	TPA-4	CASTEP	−2.37	−228.19	[66]
ZnTiO_3(H)_ (101)	MB	VASP	−1.31	−126.76	[63]
ZnTiO_3(SP)_ (101)	MB	VASP	−2.92	−282.05	[63]
ZnTiO_3_ (101)	s-Cu-TTC	VASP	−3.07	−296.56	This study

## Data Availability

Data are contained within the article and Supplementary Materials.

## Supplementary Materials

The following supporting information can be downloaded at: https://www.mdpi.com/article/10.3390/ma15093252/s1. Table S1: Bader’s charge analysis for the *s*-Cu-TTC complex before and after adsorption on the ZTO surface; Table S2: Bader’s charge analysis for the *s*-Cu-TTC complex before and after adsorption on the TO surface
