# Supplementary material for: Cu(C3H3N3S3)3 Adsorption onto ZnTiO3/TiO2 for Coordination-Complex Sensitized Photochemical Applications
Source: Materials (Basel). 2022 Apr 30;15(9):3252. doi: 10.3390/ma15093252 (PMC9100386; doi:10.3390/ma15093252)
Supplement: Supplementary file 1 [file materials-15-03252-s001.zip › materials-1676327-supplementary.pdf]

materials

MDPI

Supplementary materials

$[Cu(C_3H_3N_3S_3)_3]$ adsorption onto $ZnTiO_3/TiO_2$ for coordination-complex sensitized photochemical applications

Ximena Jaramillo-Fierro [1,*], Karol Hernández[2] and Silvia González[1]

[1] Departamento de Química, Facultad de Ciencias Exactas y Naturales, Universidad Técnica Particular de Loja, San Cayetano Alto, Loja 1101608, Ecuador; sgonzalez@utpl.edu.ec

[2] Ingeniería Química, Facultad de Ciencias Exactas y Naturales, Universidad Técnica Particular de Loja, San Cayetano Alto, Loja 1101608, Ecuador; kyhernandez@utpl.edu.ec

* Correspondence: xvjaramillo@utpl.edu.ec; Tel.: +593-7-3701444

Abstract: Currently, the design of highly efficient materials for photochemical applications remains a challenge. In this study, an efficient sensitized semiconductor was prepared, based on a coordination complex (Cu-TTC) of Cu(I) and trithiocyanuric acid on $ZnTiO_3/TiO_2$ (ZTO/TO). The Cu-TTC/ZTO/TO composite was prepared by the solvothermal method at room temperature. The structural, optical, and electrochemical characteristics, as well as the photocatalytic performance of the composite were experimentally and computationally studied. The results show that the Cu-TTC/ZTO/TO composite efficiently extended its photoresponse in the visible region of the electromagnetic spectrum. The electrochemistry of the proposed tautomeric architecture (*s*-Cu-TTC) clearly reveals the presence of metal ligand charge-transfer (MLCT) and $\pi \rightarrow \pi^*$ excitations. The maximum methylene blue (MB) dye photodegradation efficiency of 95% in aqueous solutions was achieved under the illumination of simulated solar light. Finally, computational calculations based on the Density Functional Theory (DFT) method were performed to determine the electronic properties of the *s*-Cu-TTC tautomeric structure and clarify the adsorption mechanism of this complex on the surface (101) of both $ZnTiO_3$ and TiO_2 oxides. The results obtained allow us to suggest that the Cu-TTC complex is an effective semiconductor photosensitizer and that the Cu-TTC/ZTO/TO composite can be used efficiently for photochemical applications.

Keywords: Photosensitization; Semiconductors; Coordination-polymers; Photocatalysis; Adsorption; DFT.

Citation: Lastname, F.; Lastname, F.; Lastname, F. Title. *Materials* **2022**, *15*, 3252. https://doi.org/10.3390/ma15093252

Academic Editor: Stefano Lettieri

Received: 31 March 2022
Accepted: 27 April 2022
Published: 30 April 2022

Publisher's Note: MDPI stays neutral with regard to jurisdictional claims in published maps and institutional affiliations.

Copyright: © 2022 by the authors. Licensee MDPI, Basel, Switzerland. This article is an open access article distributed under the terms and conditions of the Creative Commons Attribution (CC BY) license (https://creativecommons.org/licenses/by/4.0/).

Table S1. Bader's charge analysis for the *s*-Cu-TTC complex before and after adsorption on the ZTO surface.

	Cu-TTC				Cu-TTC adsorbed on $ZnTiO_3$			
**Atom**	**X**	**Y**	**Z**	**Charge (-*e*)**	**X**	**Y**	**Z**	**Charge (-*e*)**
Cu	6,1995	12,6505	11,8513	0,4762	5,0122	12,8559	11,8295	0,4816
C	4,7386	9,2244	8,9252	0,7881	4,0189	9,4358	8,7462	1,0936
C	5,4769	9,5762	11,2342	1,0724	4,1197	9,8553	11,1273	1,2001
C	4,9844	7,2963	10,4656	0,8033	3,4711	7,6374	10,2578	0,6239
C	5,7445	14,1285	14,7358	1,0700	4,4097	14,6245	14,5893	1,0261
C	4,3415	12,8575	16,2905	0,8027	2,8107	13,6089	16,1536	0,7198
C	5,3616	15,0081	16,9959	0,8040	3,8516	15,7953	16,6814	0,7382
C	4,2590	16,6277	10,2317	0,7729	3,2100	16,5765	9,6807	0,7492
C	5,4659	14,6511	9,4328	1,0814	5,0541	14,9872	9,4423	1,2721
C	4,7222	16,3380	7,8111	0,8269	5,0666	16,9453	8,1665	1,3460
N	5,1462	10,0181	9,9809	-3,1456	4,2355	10,2669	9,8365	-3,0464
N	5,3418	8,2208	11,4278	-3,1107	3,7359	8,5649	11,2884	-3,0257
N	4,7071	7,8718	9,2344	-3,0473	3,6477	8,1671	8,9926	-3,1866
N	4,9680	13,0505	15,0726	-3,1483	3,5459	13,6385	14,9698	-3,0933
N	5,8902	15,0751	15,7220	-3,1088	4,5193	15,6700	15,4674	-3,0373
N	4,6046	13,8641	17,2090	-3,0598	3,0145	14,7258	16,9437	-3,0669
N	4,9151	15,4261	10,4177	-3,1670	3,8997	15,4185	10,0294	-3,0538
N	4,2226	17,0316	8,9026	-3,0730	3,8460	17,2930	8,6741	-3,1102
N	5,3132	15,1364	8,1547	-3,0744	5,6472	15,7915	8,5372	-2,9234
H	5,5625	7,8464	12,3472	0,9998	3,6292	8,2067	12,2359	0,9998
H	4,4335	7,2402	8,4867	0,9999	3,4760	7,5616	8,0584	1,0000
H	6,4444	15,9009	15,5098	0,9999	5,1455	16,4336	15,2200	0,9998
H	4,1791	13,7637	18,1262	0,9999	2,4976	14,7601	17,8195	0,9997
H	3,7697	17,9204	8,7085	0,9999	3,2389	17,9934	8,1880	1,0000
H	5,6827	14,5821	7,3861	0,9999	6,6330	15,3670	7,4371	1,0000
H	5,2084	11,0273	9,7994	1,0000	4,5170	11,2409	9,6586	1,0000
H	5,0324	15,1090	11,3876	1,0000	3,4500	14,8394	10,7355	1,0000
H	4,8236	12,3297	14,3630	1,0000	3,4286	12,8429	14,3419	1,0000
S	4,9002	5,6820	10,7523	1,2491	3,0116	6,1113	10,5808	1,4598
S	4,3407	9,8173	7,4463	1,2465	4,2239	10,0875	7,2113	1,0563
S	6,0229	10,5594	12,4977	0,9911	4,4292	10,8291	12,4773	1,0061
S	3,5992	17,5010	11,4562	1,2732	1,8095	17,0136	10,3956	1,3587
S	4,6228	16,8832	6,2663	1,2227	5,8879	18,0536	7,1246	0,9092
S	6,2840	13,1967	9,6969	0,9795	5,7689	13,4899	9,8178	1,0351
S	3,3878	11,5631	16,6199	1,2430	1,8178	12,3753	16,5551	1,3597
S	5,6032	16,1652	18,1346	1,2350	4,0494	17,0733	17,6803	1,3255
S	6,4910	14,3449	13,2374	0,9977	5,2960	14,6242	13,1698	1,1465

Table S2. Bader's charge analysis for the *s*-Cu-TTC complex before and after adsorption on the TO surface.

	Cu-TTC				Cu-TTC adsorbed on TiO_2			
**Atom**	**X**	**Y**	**Z**	**Charge (-*e*)**	**X**	**Y**	**Z**	**Charge (-*e*)**
Cu	9,4704	5,6879	30,8735	0,5304	9,4588	5,6556	30,8773	0,4906
C	5,8193	7,3442	27,1964	0,7382	5,8183	7,3369	27,1985	0,8069
C	7,5555	7,0506	28,9194	1,0277	7,5534	7,0503	28,9075	1,0223
C	6,1644	5,1035	28,1778	0,7246	6,1244	5,1021	28,1319	0,7672
C	8,4281	4,7429	36,9668	0,6718	8,4277	4,7423	36,9657	0,6342
C	9,5579	5,3767	34,8685	1,0764	9,5574	5,3767	34,8681	1,1036
C	10,5849	5,9955	37,0637	0,6608	10,5843	5,9966	37,0620	0,6191
C	11,5535	4,9887	28,8098	1,1766	11,5543	4,9878	28,8064	1,2086
C	13,3878	5,2766	27,1957	0,6712	13,3908	5,2763	27,1994	0,8459
C	12,9424	7,0000	28,8177	0,8195	12,9437	7,0016	28,8133	0,8379
N	6,8613	7,7883	28,0194	-3,2802	6,8613	7,7895	28,0182	-3,1270
N	7,2094	5,6572	28,8921	-3,1862	7,1260	5,6859	28,9020	-2,9982
N	5,4753	5,9976	27,3065	-3,1343	5,4732	6,0010	27,3014	-3,0964
N	8,5976	4,7438	35,5631	-3,2973	8,5976	4,7439	35,5619	-3,0558
N	9,4684	5,3883	37,6086	-3,2043	9,4681	5,3880	37,6082	-3,0956
N	10,5215	5,9413	35,6440	-3,2045	10,5202	5,9403	35,6421	-3,0171
N	12,2612	4,6034	27,6957	-3,1629	12,2430	4,6063	27,6927	-3,0243
N	11,9053	6,1837	29,2789	-3,2512	11,9035	6,1816	29,2779	-3,1483
N	13,6263	6,5350	27,7333	-3,2276	13,6248	6,5351	27,7332	-3,1600
H	7,7437	5,0022	29,4497	1,0000	7,6795	5,0049	29,4536	1,0000
H	4,6698	5,6714	26,7491	0,9983	4,6588	5,7056	26,7403	0,9999
H	9,4663	5,3709	38,6168	0,9969	9,4641	5,3708	38,6159	0,9996
H	11,3190	6,3545	35,1816	0,9991	11,3187	6,3546	35,1811	0,9998
H	11,5538	6,4480	30,1806	1,0000	11,5530	6,4479	30,1793	1,0000
H	14,3117	7,1722	27,2830	0,9992	14,3105	7,1725	27,2818	1,0000
H	11,9647	3,7330	27,2559	0,9992	11,9755	3,7408	27,2307	1,0000
H	7,9117	4,2847	34,9961	0,9993	7,9083	4,2850	34,9955	0,9999
H	7,0201	8,7864	28,0039	0,9994	7,0205	8,7868	28,0004	0,9996
S	5,7459	3,5288	28,3706	1,4720	5,7452	3,5153	28,2070	1,3089
S	4,9991	8,4053	26,2379	1,4779	4,9240	8,4395	26,3761	1,2382
S	8,5889	7,6456	30,1435	1,1152	8,6957	7,6098	30,0026	1,1275
S	10,4230	4,0374	29,6508	1,0545	10,4151	4,0308	29,6257	1,0450
S	13,3179	8,3996	29,6170	1,4126	13,2949	8,4029	29,6103	1,2604
S	14,4407	4,5581	26,1710	1,3928	14,7087	4,4736	26,5548	1,1027
S	9,6628	5,5281	33,2085	0,9870	9,6403	5,5339	33,2046	1,0479
S	11,8138	6,6276	37,9439	1,4366	11,8054	6,6864	37,8858	1,4088
S	7,2141	3,9555	37,7289	1,5112	7,2083	3,9557	37,7063	1,3978
